# AAPM Medical Physics Practice Guideline 18.a: Superficial radiation therapy using low‐energy kilovoltage x‐rays

**DOI:** 10.1002/acm2.70857

**Published:** 2026-10-30

**Authors:** Yongsook C. Lee, Seng Boh Lim, Peter M. Mondalek, Sergio O´Reilly, David G. Saez, Ren‐Dih Sheu, Gerald A. White, Strahinja Stojadinovic

**Affiliations:** ^1^ Department of Radiation Oncology Herbert Wertheim Cancer Institute, Baptist Health South Florida Miami Florida USA; ^2^ Department of Oncological Sciences Herbert Wertheim College of Medicine, Florida International University Miami Florida USA; ^3^ Department of Medical Physics Memorial Sloan‐Kettering Cancer Center New York New York USA; ^4^ PMM Inc. Atlanta Georgia USA; ^5^ Department of Radiation Oncology Miami VA Medical Center Miami Florida USA; ^6^ Radiation Oncology SunState Medical Specialists Miami Florida USA; ^7^ Department of Radiation Oncology Mount Sinai Medical Center New York New York USA; ^8^ Colorado Associates in Medical Physics Colorado Springs Colorado USA; ^9^ Department of Radiation Oncology University of Texas Southwestern Medical Center Dallas Texas USA

**Keywords:** low‐energy kV x‐rays, medical physics practice guidelines, non‐melanoma skin cancer, superficial radiation therapy

## Abstract

Superficial radiation therapy (SRT), utilizing low‐energy kilovoltage (kV) x‐rays typically in the 50–100 kVp range, is a well‐established modality in dermatology and radiation oncology clinics for the management of non‐melanoma skin cancer and various benign dermatological conditions. The comprehensive medical physics practice guidelines presented in this report are designed to facilitate adherence to minimum standards for SRT delivery. A technical overview of commercially available SRT systems in the United States is provided. Detailed guidelines for commissioning and routine quality assurance are presented, specifying daily, monthly, and annual tests, alongside established action limits. The SRT clinical workflow is delineated, encompassing patient simulation, treatment time or monitor unit calculations, data entry, independent treatment plan review, and radiation delivery. A summary of typical state regulatory requirements for x‐ray machines is included. Adherence to relevant state regulations is emphasized and radiation safety considerations, comprising structural shielding, radiation surveys, personnel monitoring, signage, periodic spot checks, and machine operation, are discussed to ensure a safe and effective clinical environment. References to relevant societal practice guidance are provided to support standardized clinical implementation.

## 1 ABOUT MEDICAL PHYSICS PRACTICE GUIDELINES

The American Association of Physicists in Medicine (AAPM) is a nonprofit professional society whose primary purposes are to advance the science, education, and professional practice of medical physics. The AAPM has more than 10,000 members and is the principal organization of medical physicists in the United States (US).

The AAPM will periodically define new practice guidelines for medical physics practice to help advance the science of medical physics and to improve the quality of service to patients throughout the US. Existing medical physics practice guidelines (MPPGs) will be reviewed for the purpose of revision or renewal, as appropriate, on their fifth anniversary or sooner.

Each MPPG represents a policy statement by the AAPM, has undergone a thorough consensus process in which it has been subjected to extensive review, and requires the approval of the Professional Council. The MPPGs recognize that the safe and effective use of diagnostic and therapeutic radiology requires specific training, skills, and techniques, as described in each document. Reproduction or modification of the published practice guidelines and technical standards by those entities not providing these services is not authorized.

The following terms are used in the AAPM practice guidelines:

- Must and Must Not: Used to indicate that adherence to the recommendation is considered necessary to conform to this practice guideline. While must is the term to be used in the guidelines, if an entity that adopts the guideline has shall as the preferred term, the AAPM considers that must and shall have the same meaning.
- Should and Should Not: Used to indicate a prudent practice to which exceptions may occasionally be made in appropriate circumstances.

## 2 INTRODUCTION

### 2.1 Background and rationale

Superficial radiation therapy (SRT) has served for several decades as a mainstay treatment for non‐melanoma skin cancer (NMSC), including basal cell carcinoma and squamous cell carcinoma.^1^ SRT has also shown efficacy in managing various non‐cancerous dermatological conditions, such as keloid scars, mycosis fungoides, and psoriasis.^2^, ^3^, ^4^, ^5^ SRT encompasses a spectrum of modalities, including low‐energy kilovoltage (kV) x‐rays (superficial x‐rays), low‐energy electrons and high dose rate (HDR) brachytherapy utilizing either a radioactive source or a kV source.^1^, ^6^ Among these modalities, superficial x‐rays are widely implemented in dermatology and radiation oncology clinics due to their operational simplicity and cost‐effectiveness.^2^, ^7^

Despite widespread clinical adoption, there is a paucity of comprehensive guidelines for SRT utilizing low‐energy kV x‐rays. At the time of this writing, the AAPM Task Group (TG)‐61 report represents the sole AAPM guidance on SRT and its scope is limited to reference dosimetry.^8^ Similarly, earlier codes of practice endorsed by the International Atomic Energy Agency (IAEA) and the Institute of Physics and Engineering in Medicine and Biology (IPEMB) prioritized reference dosimetry.^9^, ^10^, ^11^ While the Institute of Physics and Engineering in Medicine (IPEM) Report 18 (2nd ed.) published in 2018 addresses quality control (QC) for superficial x‐ray units,^12^ and the Canadian Organization of Medical Physicists (COMP) issued guidelines in 2018 specific to QC and quality assurance (QA) of kV x‐ray therapy machines,^13^ neither comprehensively addressed the broader spectrum of considerations for SRT implementation. This report aims to bridge that gap by establishing comprehensive MPPG for SRT utilizing low‐energy kV x‐rays. These guidelines are intended to assist SRT teams in dermatology and radiation oncology clinics in establishing and maintaining SRT programs while adhering to appropriate minimum standards in routine clinical practice.

### 2.2 Scope and charges

This report focuses primarily on SRT utilizing low‐energy kV x‐rays. Established AAPM guidelines already exist for other SRT modalities, such as electrons and HDR brachytherapy including electronic brachytherapy.^14^, ^15^, ^16^, ^17^, ^18^ One can refer to those resources for further details. Some commercial kV x‐ray therapy units have both SRT and electronic brachytherapy capabilities or offer superficial and orthovoltage x‐ray energies (up to 300 kVp) in a single unit. In alignment with the AAPM TG‐61 definition of superficial x‐rays,^8^ this report discusses SRT utilizing x‐ray energies up to 100 kVp; the typical clinical energy range of SRT is 50 ‐ 100 kVp. Legacy superficial x‐ray machines still in clinical operation but no longer commercially available are excluded from this scope. The official charges of this report encompass the following:

a) Review commercially available superficial x‐ray systems in the US
b) Describe acceptance testing, commissioning, and routine QA for superficial x‐ray units
c) Delineate SRT treatment workflow and methods
d) Discuss regulatory requirements and radiation safety
e) Provide societal clinical practice recommendations

### 2.3 Intended audience

This MPPG is intended for a broad audience involved in SRT in the clinical setting. This includes qualified medical physicists (QMPs), physicians (radiation oncologist or dermatologist), qualified medical dosimetrists (QMDs) and radiation therapists (RTTs).

## 3 QUALIFICATIONS AND RESPONSIBILITIES OF PERSONNEL

Each member of the SRT team must be appropriately trained, with their specific responsibilities clearly defined. The qualifications and responsibilities of the QMP are defined in the AAPM MPPG 10.b and associated documents.^19^, ^20^, ^21^ Similarly, the American College of Radiology, in collaboration with the American Radium Society, outlines the qualifications and responsibilities of radiation oncologists, QMDs, and RTTs in their Practice Parameter for Radiation Oncology.^21^ However, a universally recognized definition for a dermatologist specifically qualified in SRT has yet to be established.

For the purposes of this report, “properly trained personnel” explicitly refers to SRT team members who have been instructed by a QMP and have demonstrated the competency to perform specific tasks under the general supervision of a QMP. Required daily and monthly QA can be performed by a QMP, an RTT, a medical physics resident, or a medical physicist assistant. Treatment time calculations can be performed by a QMP, a QMD, or a physician (e.g., dermatologist). Initial plan/chart review, weekly chart review and end‐of‐treatment (EOT) chart review can be done by a QMP, a QMD, an RTT or a physician (e.g., dermatologist).

## 4 SUPERFICIAL X‐RAY SYSTEMS

### 4.1 Treatment units

At the time of this writing, there are two commercial manufacturers supplying SRT units in the US: Sensus Healthcare, Inc. (Boca Raton, FL) and Xstrahl Ltd. (Walsall, West Midlands, UK). Pertinent characteristics of these units are summarized in Tables 1 and 2. Note that electronic brachytherapy units using a kV source (e.g., Elekta Xoft) are not included in this report. Although various tube potentials are available from the manufacturers, the clinically utilized energy range for SRT treatments is typically 50–100 kVp. Figure 1 shows examples of SRT units from both manufacturers.

**TABLE 1:** Characteristics of Sensus Healthcare units

Manufacturer		Sensus Healthcare, Inc., Boca Raton, FL, USA		
**Unit type**		SRT‐100^TM^ (SRT)	SRT‐100+ ^TM^ (Grenz + SRT)	SRT‐100 Vision ^TM^ (Grenz + SRT + eBT)
**Energy [kVp]**		50, 70, 100	Grenz: 20, 30; SRT: 50, 70, 100	Grenz/eBT: 20, 30; SRT/eBT: 50, 60, 70, 80, 90, 100
**Filter (built‐in automatic)**		3: each energy mode	4: Grenz, SRT50, SRT70, SRT100	6: Grenz, SRT50, SRT70, SRT100, Triune^TM^ Par, Triune^TM^ Distal
**Applicator diameter [cm] or field size [cm^2^]**	SRT	1.5, 2.0, 2.5, 3.0, 4.0, 5.0		
		(optional) 1.0, 7.3, 10.0, 12.7, 18.0 × 8.0		
	eBT	N/A	N/A	1.0, 1.5, 2.0, 2.5, 3.0, 3.5
**Source‐to‐skin distance [cm]**	SRT	15 for 1.0–7.3 cm applicators, 25 for 10.0–12.7 cm applicators, 30 for 18.0 × 8.0 cm^2^ applicator		
	eBT	N/A	N/A	6 for all applicators
**Imaging capability**		N/A	N/A	Ultrasound (20 MHz)
**Time‐based or MU‐based**		Time	Time	Time
**Mobile within a room**		Yes	Yes	Yes

Abbreviations: eBT, electronic brachytherapy; kVp, kilovoltage peak; MU, monitor unit; SRT, superficial radiation therapy

**TABLE 2:** Characteristics of Xstrahl units

Manufacturer	Xstrahl Ltd, Walsall, West Midlands, UK				
**Unit type**	RADiant^TM^Aura (SRT + eBT)	Xstrahl 100 (Grenz + SRT)	Xstrahl 150 (Grenz + SRT + ORT)	Xstrahl 200 (Grenz + SRT + ORT)	Xstrahl 300 (SRT + ORT)
**Energy range [kVp]**	SRT: 50, 80; eBT: 50, 70	10–100	10–150	20–220	40–300
**Filter (interlocked)**	3 (1 warm‐up & 2 clinical)	6 (1 warm‐up & 5 clinical)	10 (1 warm‐up & 9 clinical)		
**Standard applicator diameter [cm] or field size [cm^2^]**	1.0, 1.5, 2.0, 3.0, 4.0	2.0, 4.0, 6.0, 8.0, 10.0, 15.0	1.5, 2.0, 2.5, 3.0, 4.0, 5.0, 10.0, 15.0	3.0, 4.0, 5.0, 10.0, 4.0 × 4.0, 6.0 × 6.0, 8.0 × 8.0, 10.0 × 10.0, 15.0 × 15.0, 20.0 × 20.0	
		(optional) custom field sizes available at request (e.g., 18 cm, 50.0 × 50.0 cm^2^)			
**Focus‐to‐skin distance [cm]**	SRT: 6 for 1.0–4.0 cm applicators eBT: 5 for 1.0–4.0 cm applicators	15 for 2.0–8.0 cm applicators	15 for 1.5–5.0 cm applicators	20 for 3.0–10.0 cm applicators	30 for 3.0–5.0 cm applicators
		25 for 10.0–15.0 cm applicators	25 for 10.0–15.0 cm applicators	50 for 4.0 × 4.0–20.0 × 20.0 cm^2^ applicators	50 for 4.0 × 4.0–20.0 × 20.0 cm^2^ applicators
**Time‐based or MU‐based**	Time	Time	Time or MU	Time or MU	Time or MU
**Mobile within a room**	Yes	Yes	No	No	No

Abbreviations: eBT, electronic brachytherapy; kVp, kilovoltage peak; MU, monitor unit; ORT, orthovoltage radiation therapy; SRT, superficial radiation therapy

**FIGURE 1:**
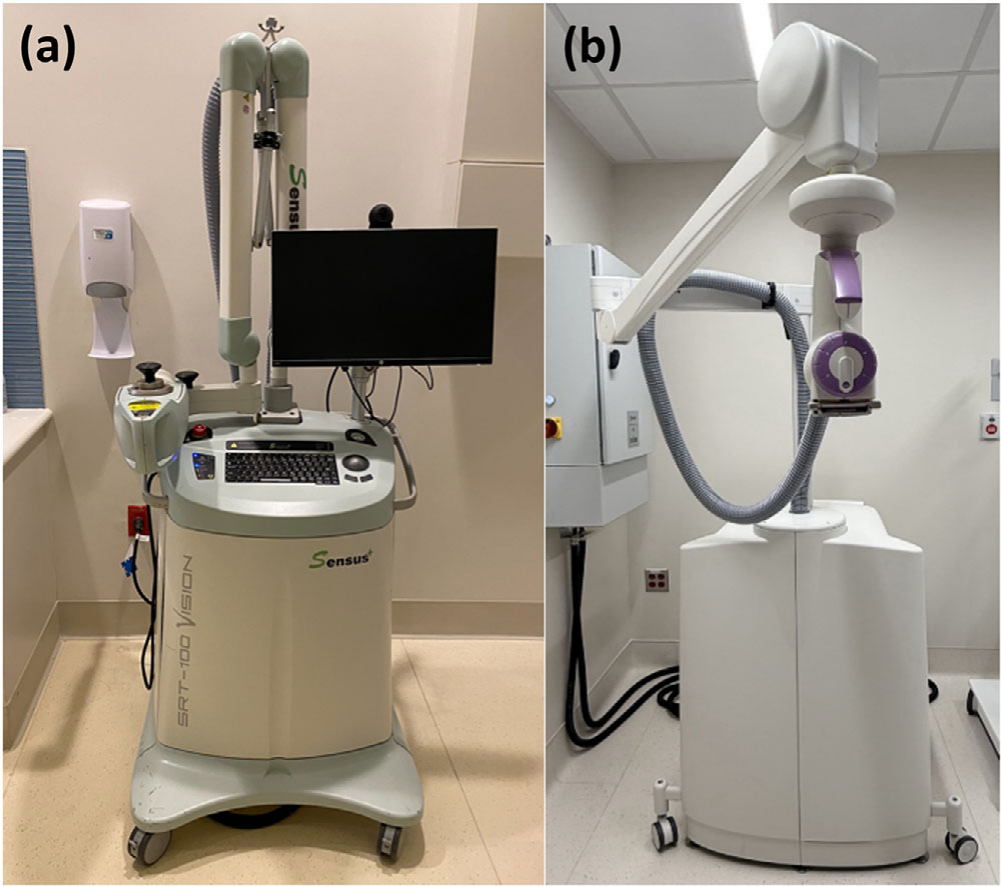
Examples of superficial x‐ray units: (a) Sensus Healthcare SRT‐100 Vision and (b) Xstrahl 100

### 4.2 Clinical interface

A clinical interface (CI) is available for both manufacturers’ systems to facilitate communication with treatment units. Patient information and treatment parameters are entered/selected and saved directly within CI. Unlike the Sensus Healthcare interface, the Xstrahl CI can communicate with third‐party Radiation Oncology Electronic Medical Record (RO‐EMR) systems, such as Aria (Varian Medical Systems, Palo Alto, CA) or Mosaiq (Elekta AB, Stockholm, Sweden) via the XBridge software. Depending on the RO‐EMR system used, either a subset or all of the information saved in the Xstrahl CI is transferred (data synchronization).

## 5 ACCEPTANCE, COMMISSIONING AND ROUTINE QA OF SUPERFICIAL X‐RAY UNIT(S)

Recommended minimum practices for acceptance testing, commissioning, and routine QA of a superficial x‐ray unit are listed in Table 3. Additionally, the table includes QA equipment recommended for these procedures.

**TABLE 3:** Recommended minimum practice standards for superficial x‐ray unit(s)

Acceptance testing		QA equipment
Manufacturer acceptance testing procedures		Per manufacturer
**Commissioning**		**QA equipment**
Radiation survey		Calibrated survey meter
Machine	Necessary data for clinical treatments	
	Beam quality: HVL (all beam energy/filter combinations intended for clinical use)	Ionization chamber, electrometer, aluminum plates
	Applicator factors (all beam energy/filter combinations/all applicators intended for clinical use)	Ionization chamber, electrometer
	Absolute output calibration (AAPM TG‐61 protocol or an equivalent standard) (all beam energy/filter combinations/reference applicator of the same FSD/SSD or all applicators intended for clinical use)	Calibrated ionization chamber, electrometer
	PDD (all beam energy/filter combinations/all applicators intended for clinical use)	Ionization chamber, radiochromic films or solid state detectors, water tank or solid water slabs, electrometer
	Profiles (all beam energy/filter combinations/all applicators intended for clinical use)	Ionization chamber, radiochromic films or solid state detectors, water tank or solid water slabs, electrometer
	Congruence between radiation field and applicator size at FSD/SSD (all beam energy/filter combinations/all applicators intended for clinical use)	Radiochromic films
	Inverse square law validation with an air gap if applicable	Ionization chamber, electrometer
	Focal spot size	Pinhole or resolution tool, radiochromic film
Software	Treatment time/MU calculation worksheet, look up table, TPS or commercial/in‐house software validation	N/A
Connectivity between treatment unit CI and RO‐EMR system if applicable		N/A
Routine QA tests/basline measurements, action limits and QA documentation		See routine QA

Routine QA			
Frequency	Test	Action limit	QA equipment
Daily or day of treatment	Auto warm‐up including self‐check if applicable	Complete	N/A
	Safety/interlocks: audio/visual monitors, door interlock, beam‐on indicator, beam‐off at key‐off, beam interrupt, filter interlock (scheduled filter/applicator combinations)	Functional	N/A
	kVp‐filter correlation (scheduled beam energy/filter combinations)	Functional	N/A
	Output constancy with head straight (scheduled beam energy/filter combinations/an applicator)	Baseline ±3%	Ionization chamber or diode, electrometer
	Backup timer (time‐based systems)/Backup MU (MU‐based systems)	±2%	N/A
	Ultrasound: Functional check (Sensus SRT‐100 Vision unit only)	Functional	N/A
Monthly	Output constancy with head straight (all beam energy/filter combinations/an applicator in clinical use)	Baseline ±3%	Ionization chamber or diode, electrometer
	Timer accuracy (time‐based systems) (range of 10 – 1000 cGy)	±2% or ±0.02 min	Calibrated stopwatch
	Mechanical stability and safety	Functional	N/A
	Filter interlock (Xstrahl units only) (all filter/applicator combinations in clinical use)	Functional	N/A
	Applicator integrity and indicators (all applicators in clinical use)	Functional	N/A
Annual	Beam quality: HVL (all beam energy/filter combinations/a small applicator in clinical use)	Baseline ±10%	Ionization chamber, electrometer, Aluminum sheets
	Absolute output calibration (all beam energy/filter combinations/all applicators in clinical use)	±3.5%^†^ of the current output used clinically	Calibrated ionization chamber, electrometer
	Output reproducibility (beam energy/filter combinations/an applicator in clinical use)	2% (standard deviation/mean×100%)	Ionization chamber, electrometer
	Output error associated with beam interrupt (beam energy/filter combinations/an applicator in clinical use)	±2% of output without beam interupt	Ionization chamber, electrometer
	Output constancy with varying head tilt and rotation (all beam energy/filter combinations/an applicator in clinical use)	±2% of output with head straight	Ionization chamber, electrometer
	Output consistency vs. dose rate (MU‐based systems) (all beam energy/filter combinations/an applicator in clinical use)	±2% of output for reference dosimetry dose rate	Ionization chamber, electrometer
	Output linearity (time‐based systems) (beam energy/filter combinations/an applicator in clinical use) (range of 10 – 1000 Gy)	±1% of nominal	Ionization chamber, electrometer
	MU linearity (MU‐based systems) (all beam energy/filter combinations/an applicator in clinical use) (range of 10 – 1000 Gy)	±2% of nominal	Ionization chamber, electrometer
	Timer end‐effect error (time‐based systems) (beam energy/filter combinations/an applicator in clinical use)	±0.01 min	Ionization chamber, electrometer
	Applicator factors (beam energy/filter combinations/all applicators in clinical use)	Baseline ±2%	Ionization chamber, electrometer
	PDD verification^§^ (all beam energy/filter combinations/all applicators in clinical use): spot checks	Baseline ±3%	Ionization chamber, radiochromic films or solid state detectors, water tank or solid water slabs, electrometer
	Profiles verification (lowest beam energy/filter combination/largest applicator in clinical use): x‐ray field uniformity	Baseline ±5%	Ionization chamber, radiochromic films or solid state detectors, water tank or solid water slabs, electrometer
	Congruence between radiation field and applicator size at FSD/SSD (beam energy/filter combinations/all applicators in clinical use)	±2 mm	Radiochromic films
	Focal spot size	0%–30% of nominal size^33^	Pinhole or resolution tool, radiochromic film
	Records	Complete	N/A
	Emergency procedures	Complete	N/A
Upgrade/Repair/Service/Relocation	Manufacturer recommended testing	As recommended	See routine QA
	Verify/Reestablish QA baselines	As needed	See routine QA

^†^ Combined standard uncertainty for the dose rate to water at the water surface using the in‐air method (AAPM TG‐61 report Table III)^3^

^§^ Applicable only to clinics/institutions using PDD clinically

*Baselines established at the time of commissioning.

Abbreviations: AAPM, American Association of Physicists in Medicine; CI, clinical interface; FSD, focus‐to‐skin distance; HVL, half value layer; kVp, kilovoltage peak; MU, monitor unit; PDD, percent depth dose; QA, quality assurance; RO‐EMR, radiation oncology electronic medical record; SSD, source‐to‐skin distance; TG, task group; TPS, treatment planning system.

### 5.1 Acceptance testing

In instances where a manufacturer offers or mandates acceptance testing, the process must be conducted by vendor's representative under the oversight of an independent QMP.^19^ Consistent with protocols for diagnostic imaging units and linear accelerators,^22^, ^23^ it is recommended that the acceptance testing report be co‐signed by both the vendor representative and the QMP and kept on file for future reference.

### 5.2 Commissioning

SRT commissioning is primarily focused on the treatment delivery machine, as SRT procedures typically do not require immobilization devices or ancillary systems for imaging and motion management. For units equipped with integrated imaging, such as the Sensus Healthcare SRT‐100 Vision, the ultrasound system must be properly commissioned.^24^ Relevant ultrasound testing protocols can be found in the AAPM TG‐128 report.^25^ Furthermore, for SRT treatment planning systems (TPS), if applicable, users can benefit from the general guidance provided in the AAPM TG‐53 report regarding acceptance testing, commissioning procedures, routine QA practices, and ongoing monitoring of the treatment planning process.^26^

As a standard practice, commissioning measurements must be performed by a QMP.^19^ Depending on the jurisdiction, this practice can also be mandated by state regulations. Table 3 details the minimum required commissioning data. Prior to initiating commissioning activities, a comprehensive radiation survey in the vicinity of the treatment room is mandatory.^27^ Section 7.2 provides a detailed discussion of the radiation survey. Half value layer (HVL) measurements for each energy level shall be performed before absolute output calibration. AAPM TG‐61 requires a detector with limited beam‐quality dependence (within 5%)^8^ to ensure that spectral changes from beam hardening do not distort HVL measurements. HVL is measured not only to verify beam quality but also to interpolate air‐kerma calibration factors, mass energy‐absorption coefficients and backscatter factors used for absolute output calibration in the AAPM TG‐61 protocol.^8^ Values derived from HVL measurements must be used instead of vendor provided values. The methodology (in‐air method) and detector selection recommended in the AAPM TG‐61 report, or an equivalent standard, must be followed for absolute output calibration.^8^, ^9^, ^10^, ^11^ Absolute output calibration for all energy/filter combinations intended for clinical use can be performed either for the reference applicator at the specified focus‐to‐skin distance (FSD) for Xstrahl units or source‐to‐skin distance (SSD) for Sensus Healthcare units, with applicator factors measured and applied to other applicators, or individually for all applicators, thereby eliminating the need for applicator factors. In accordance with standard dosimetry protocols, absolute output calibration requires an ionization chamber with a calibration factor traceable to an Accredited Dosimetry Calibration Laboratory.^8^ The central axis percent depth dose (PDD) data must be acquired for all energy/filter combinations and all applicators intended for clinical use. Regardless of the prescription depth, measured PDDs can be clinically used for beam energy selection. In scenarios where it is not possible to directly measure the PDDs, the AAPM TG‐61 report recommends interpolating from the published data in the British Journal of Radiology Supplement 25.^8^, ^28^ Alternatively, readers can refer to Xiong et al.^2^ for more recent high‐resolution solid‐state detector PDD curves measured in water. For Sensus Healthcare units, however, direct measurement and verification of PDDs are recommended due to potential inter‐unit variability.^7^ These measurements must be performed using the same QA equipment and setup described in the relevant literature.^7^, ^24^ Measured PDDs must be referenced to device‐specific published PDDs within a tolerance of ±3%. Specifically, for Sensus Healthcare units, acquiring PDD data using a water tank is challenging due to the unit's x‐ray tube design; therefore, PDDs are typically measured using solid water slabs. When this approach is employed, the use of proper water‐equivalent slabs (which mimic liquid water to provide radiologically equivalent scattering and attenuation) is essential as certain commercial solid water slabs introduce non‐negligible PDD discrepancies in the low‐energy x‐ray range compared to liquid water.^29^ Additionally, the inherent uncertainties associated with PDD measurements using solid water slabs, as reported by Lee et al.,^30^ must be evaluated. For low‐energy kV x‐ray PDD measurements, it is recommended to use an ionization chamber that exhibits a flat energy response at depth, thereby minimizing the need for depth‐dependent correction factors.^8^, ^31^ If a suitable ionization chamber is unavailable, radiochromic films offer a viable alternative, having demonstrated agreement with ionization chambers within 4% for energies ≤100 kVp.^7^, ^32^ Furthermore, modern solid‐state detectors, such as diamond and silicon detectors, are reliable options for low‐energy kV x‐ray PDD measurements, as recently reported by Xiong et al.^2^

Along with the data collected for patient treatments, the following commissioning tasks are recommended. While beam profile measurements are not clinically used for treatment time/monitor unit (MU) calculations, inline and crossline profiles must be measured to evaluate x‐ray field uniformity, with particular attention to the heel effect in the anode‐cathode direction. Beam profile measurements must be performed at commissioning for all energy/filter combinations and all applicators intended for clinical use. Periodically thereafter, routine spot checks should assess the most frequently used energy/filter combinations as well as extreme combinations, such as the lowest energy and largest applicator. When applicable, these checks should include points at the prescription depth to verify adequate target coverage. At a depth of 5 mm or less (typical treatment depth for SRT),^6^ the field uniformity in both the anode‐cathode and its perpendicular directions should be within 10% across 80% of the applicator's dimensions.^7^, ^24^ Neither manufacturer's system incorporates a light field. Nonetheless, the congruence between radiation field and applicator size at the FSD/SSD must be verified. If a clinical setup includes an air gap between the applicator and the patient's skin, the inverse square law must be validated. It is recommended using the smallest applicator for inverse square law validation, as a 4% difference was reported for a 2.5 cm applicator at 10 cm FSD.^11^ The focal spot size should be measured to verify agreement with the manufacturer's nominal specification or the provided factory test data baseline.^12^, ^13^, ^33^ If an RO‐EMR system is employed, verifying the connectivity between the unit's CI and the RO‐EMR system, along with the integrity of transferred data, is crucial. Concurrently, routine QA tests/baseline measurements for all energy/filter combinations/all applicators (if applicable) intended for clinical use, action limits and QA documents must be established.^34^

Whether commercial TPS or in‐house software/tool is used as a primary or secondary system, the system for treatment time/MU calculations must be fully commissioned and validated before clinical use. Based on data collected during commissioning, a treatment time/MU calculation worksheet, or a lookup table must be created and verified. For a TPS (e.g., Xstrahl XBeam) or commercial software (e.g., ClearCalc; Radformation, Inc., New York, NY) supporting an SRT module,^35^, ^36^ all vendor‐required physics data (e.g., dose rates, PDDs) must be entered into the software and verified. The resulting treatment time or MU calculations are then systematically validated through independent hand calculations, cross‐method comparisons, or both. Any in‐house software must be commissioned in a similar fashion, with all validation steps formally documented.

Upon completion of commissioning a unit and/or TPS/software, a comprehensive end‐to‐end test of the newly implemented workflow, involving all relevant personnel and encompassing setup simulation through treatment delivery using a phantom, is recommended.^24^ This test aims to identify any potential weaknesses in the overall treatment process.^24^ Additionally, independent verification of reference dosimetry for all applicable beam energies established during commissioning is recommended prior to clinical use. For example, UT MD Anderson Radiation Dosimetry Services offers independent output calibration verification for low‐energy x‐rays (HVL ≥1.9 mm Al). Finally, a comprehensive commissioning report detailing the work performed and key findings must be documented for future reference.

### 5.3 Routine QA

Recommended routine QA tests and the corresponding action limits are listed in Table 3. These QA tests and action limits are established primarily based on the guidelines outlined in the 2008 IAEA report, IPEM Report 81 (2nd ed.), and COMP report.^12^, ^13^, ^34^ All QA results must be documented and reviewed independently by a QMP, or by a radiation safety officer (RSO) of the facility.

#### 5.3.1 Daily QA

Daily QA is performed by properly trained personnel. Safety and interlock systems, as well as selected energy‐filter pairings, must be checked daily before treatment. Daily output checks must be performed for at least the beam energies used that day. An appropriate detector (e.g., an ionization chamber) can be used for daily output constancy checks. Alternatively, a vendor‐provided device may be used for correct energy/filter combinations. An example is the RADCheck™ diode from Sensus Healthcare for SRT‐100 units, with baseline values for all commissioned energy/filter combinations configured directly at the machine.^37^ A baseline value for a daily QA detector must be established immediately following absolute output calibration. Additionally, backup timer (time‐based systems) or backup MU (MU‐based systems) must be checked daily. For systems with an imaging component, such as an SRT‐100 Vision unit, a functional check of ultrasound must be performed if ultrasound is to be used that day.

#### 5.3.2 Monthly QA

Monthly QA is performed by properly trained personnel. In clinics with limited staffing and QA tools, monthly QA can be an extension of daily QA, focusing on additional checks. Monthly output verification must encompass all clinically used energy/filter combinations. If a detector other than the daily QA detector is used for output constancy checks, a new baseline for that detector must be established immediately following absolute output calibration. For time‐based systems, timer accuracy must be compared to a calibrated stopwatch or timer. One can refer to the National Institute of Standards of Technology recommended practice guide for stopwatch and timer calibrations.^38^ Additionally, monthly QA must include verification of the mechanical stability and safety of the unit and its accessories, filter interlock functionality, as well as the integrity of applicators and their associated indicators.

#### 5.3.3 Annual QA

Annual QA is performed by or under the direct supervision of a QMP, either internal or external to the clinic, using appropriate QA tools (detector(s), films, electrometer, and/or solid water slabs/water tank).^19^ Beam quality (HVL) and absolute output calibration for all clinically used energy/filter combinations must be verified annually following the AAPM TG‐61 protocol or an equivalent standard. At the QMP's discretion, output adjustments can be made when measurements reach the set action limit. Otherwise, adjustments are mandatory for deviations exceeding ±5% from the outputs currently in clinical use. In such cases, the unit console (if necessary), treatment time/MU calculation worksheet, lookup table, TPS and/or commercial/in‐house software, must be updated to reflect the new values. Annual verification of output reproducibility, output error associated with beam interrupt and output constancy for varying gantry/head tilt and rotation angles is required. For MU‐based systems, output constancy at various dose rates must be evaluated. Output linearity for time‐based systems or MU linearity for MU‐based systems must be verified annually. In conjunction with output linearity measurements, a timer end‐effect error must be assessed for time‐based systems. The timer end‐effect error may be determined graphically by extrapolating a timer linearity line or by using the equation described by Attix.^8^ It is recommended to verify applicator factors on an annual basis. Inconsistent values of applicator factors could be indicative of physical damage to the applicator(s). Annual PDD spot checks are recommended if PDD data are used clinically, i.e., for non‐zero prescription depths. Annual PDD measurements must be performed using the same QA equipment and setup as during commissioning and must be referenced to the baseline data established at that time. A PDD measurement and verification are required if the HVL changes by more than ±10% relative to the baseline. At a minimum, annual beam profile measurements are required for the most frequently used energy/filter combinations as well as extreme combinations, such as the lowest energy and largest applicator. During these measurements, x‐ray field uniformity must be verified to remain within ±5% of the commissioning baseline. If applicable, additional measurements must be performed at clinically relevant prescription depths to ensure consistent target coverage.^13^, ^34^ The congruence between radiation field and applicator size at the specified FSD/SSD is warranted annually. The focal spot size should be measured annually to verify consistency over time.^13^ Documentation regarding daily QA, monthly QA, preventive maintenance inspection (PMI), service calls and post‐service QA must be completed and reviewed at least annually. Annual emergency training or an in‐service session conducted by a QMP, a trained individual or an RSO of the facility is recommended. For SRT‐100 Vision units, periodic vendor PMI may include ultrasound QA. Otherwise, it is recommended that the guidelines outlined in the AAPM TG‐128 report be followed for relevant QA testing.^25^

## 6 SRT TREATMENT WORKFLOW AND METHODS

### 6.1 Treatment workflow

Unlike conventional radiation therapy, SRT treatment does not require a computed tomography simulation and is typically delivered in a clinical setup. Figure 2 illustrates common treatment workflows using superficial x‐ray units. The first treatment fraction may be delivered either on the same day as the setup simulation (Figure 2(a)) or during the next patient visit (Figure 2(b)). Note that workflow variations may occur across different clinics.^24^

**FIGURE 2:**
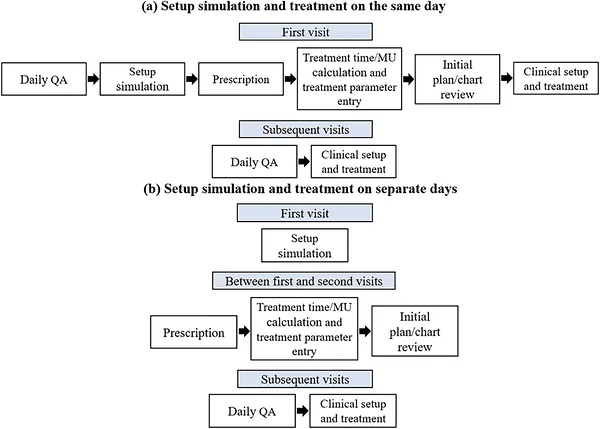
Typical superficial radiation therapy (SRT) treatment workflows for: (a) same‐day simulation and treatment, and (b) separate‐day simulation and treatment. Abbreviations: MU, monitor unit; QA, quality assurance

#### 6.1.1 Daily QA

Daily QA must be performed each day of treatment, as outlined in Table 3.

#### 6.1.2 Setup simulation

A setup simulation is performed to determine patient positioning, immobilization, and treatment delivery parameters. Optimal positioning and reproducible immobilization (e.g., a headrest for facial treatment) are essential to ensure daily setup consistency. Clinical and pathologic reports, along with physical examinations, are used to identify the lesion, which is then marked on the skin by the treating physician (Figure 3). Applicator size, energy/filter, treatment depth (if prescribed to a non‐zero depth) and air gap (if applicable) are subsequently determined. For open‐ended applicators, the skin surface should be flush with the end of the applicator (no stand‐in or stand‐off). If this is not feasible, corresponding dosimetry calculations should be performed to inform the physician of the magnitude of dose variation across the treatment field. Appropriate shielding (e.g., lead shield) may be used to protect adjacent lesions (Figure 3) and critical structures (e.g., thyroid gland or eyes). The lead shield must be covered with plastic wrap to prevent direct skin contact and mitigate possible toxicity (Figure 3(a)). Before the simulation, patient information (name, date of birth, medical record number, and treatment site with laterality) is entered into the unit's CI and verified for accuracy. Checklist items for simulation, as recommended in MPPG 11.a, can be adapted for SRT setup simulations.^39^

**FIGURE 3:**
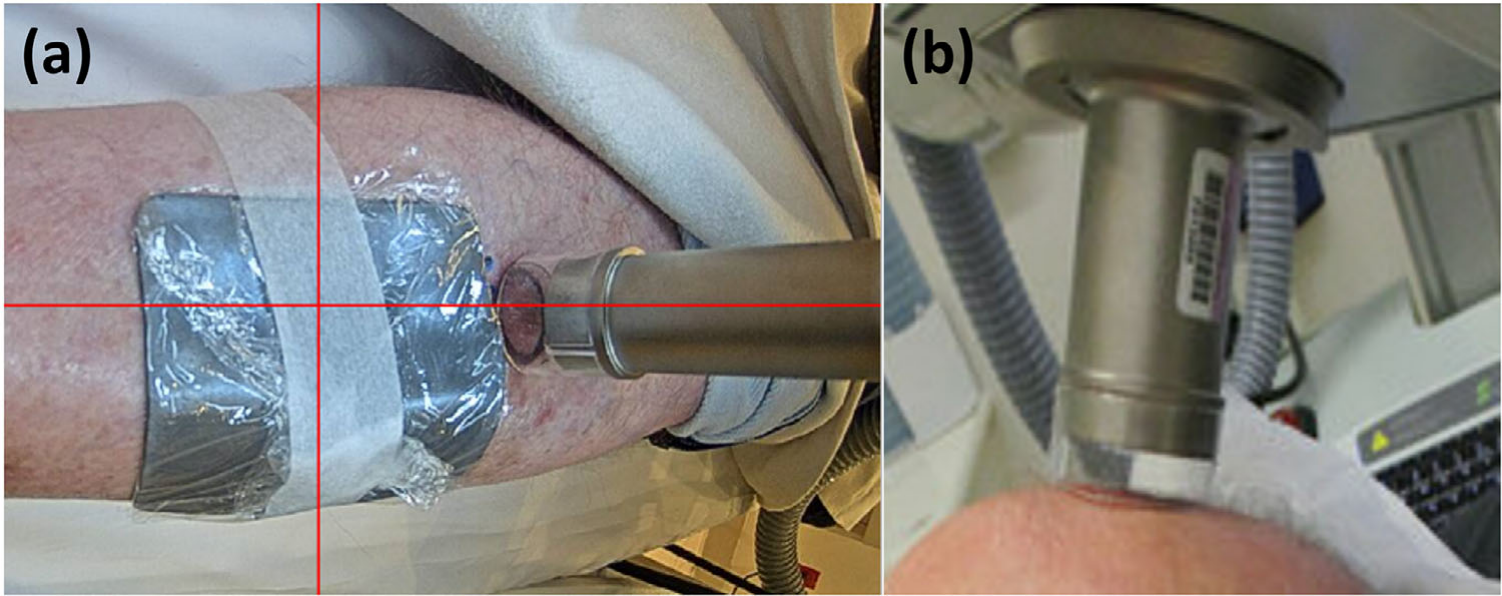
Examples of clinical setups for superficial radiation therapy (SRT) treatments: (a) leg and (b) scalp

#### 6.1.3 Prescription

The prescription must include the treatment site (anatomic site and laterality), modality, energy, filter, applicator size, fractional dose, total dose, treatment delivery pattern, prescription depth, and pre‐treatment preparation (e.g., shielding) as well as imaging technique and frequency if applicable.^39^ The prescription must be approved and signed by the treating physician before treatment.

#### 6.1.4 Treatment time or MU calculation

Based on the prescription and setup simulation notes, treatment time (time‐based systems) or MUs (MU‐based systems) must be calculated by properly trained personnel using one of the methods described below. These methods can be used for either primary calculation or secondary verification; however, both must be performed as a safety check. Upon completion of the calculation, a treatment plan document containing treatment delivery parameters and calculated treatment time/MU must be created. The plan document must be reviewed and signed by the treating physician before treatment. For patient treatments requiring a non‐standard setup, such as custom irregular fields and shielding that substantially alter backscatter, patient‐specific measurements using in vivo dosimetry are recommended to verify dosimetric accuracy.^24^

**Time‐based systems: all Sensus Healthcare and Xstrahl units**

**SRT time calculation worksheet**

A standardized clinical worksheet is essential for procedural consistency when a dedicated TPS is unavailable. Such a worksheet incorporates essential parameters, including dose rate, backscatter factors, FSD/SSD, PDDs and the time calculation equation (Figure 4). Treatment time for a selected energy/filter combination and an applicator is calculated using equation (1) below.^2^

(1) $$\begin{array}{cc} t\;\left(min\right) & =D_{Rx}\;\left(cGy\right)÷\left[{\dot{D}}_{w,z=0}\left(FSD,\;d_{FSD}\right)\left(\frac{cGy}{min}\right)\right.\\ & \;\times {\left(\frac{FSD}{FSD^{\prime }}\right)}^{2}\times \left(\frac{BSF^{\prime }\left(FSD^{\prime },\;d_{FSD^{\prime }}\right)}{BSF\left(FSD,\;d_{FSD}\right)}\right)\\ & \;\times \left(\frac{PDD\left(FSD,\;z,\;d\right)}{100\%}\right)\times \left\{{\left(\frac{FSD^{\prime }}{FSD}\right)}^{2}\right.\\ & \;\left.\left.\times {\left(\frac{FSD+z}{FSD^{\prime }+z}\right)}^{2}\right\}\right]\; \end{array}$$

where *t* is the treatment time per fraction (min), $D_{Rx}$ is the prescription dose per fraction (cGy), ${\dot{D}}_{w,z=0}$ is the dose rate to water at the water surface (cGy/min) at the specified FSD for an applicator diameter of *d_FSD_*, FSD refers to the focus‐to‐skin distance, representing the distance between the focal spot and the applicator's distal end, $FSD^{\prime }$ is defined as FSD + *g*, where the air gap *g* represents the separation between the applicator and the patient's skin, the term ${\left(\frac{FSD}{FSD^{\prime }}\right)}^{2}$ is the inverse square factor for the dose rate change at $FSD^{\prime }$, the expression $\left(\frac{BSF^{\prime }\left(FSD^{\prime },\;d_{FSD^{\prime }}\right)}{BSF(FSD,\;d_{FSD})}\right)$ is the ratio of the backscatter factors at $FSD^{\prime }\;versus\;FSD$ for the corresponding applicator diameters, $d_{FSD^{\prime }}\;\mathrm{and}\;d_{FSD}$, PDD is the percent depth dose at the prescription depth *z* for the applicator diameter *d* and the given FSD, and the term ${\left(\frac{FSD^{\prime }}{FSD}\right)}^{2}\times {\left(\frac{FSD+z}{FSD^{\prime }+z}\right)}^{2}$ is the Mayneord's F factor for the PDD change from FSD to $FSD^{\prime }$. Without an air gap (*g* = 0), $FSD^{\prime }$ is equal to FSD, and the inverse square factor, the backscatter factor ratio, and the Mayneord's F factor all become unity. When the measured timer end‐effect error exceeds the timer resolution, the timer end‐effect correction must be incorporated into Equation (1) to avoid introducing an unaccounted systematic error. Note that for Sensus Healthcare units, FSD is replaced with SSD in Equation (1).

**FIGURE 4:**
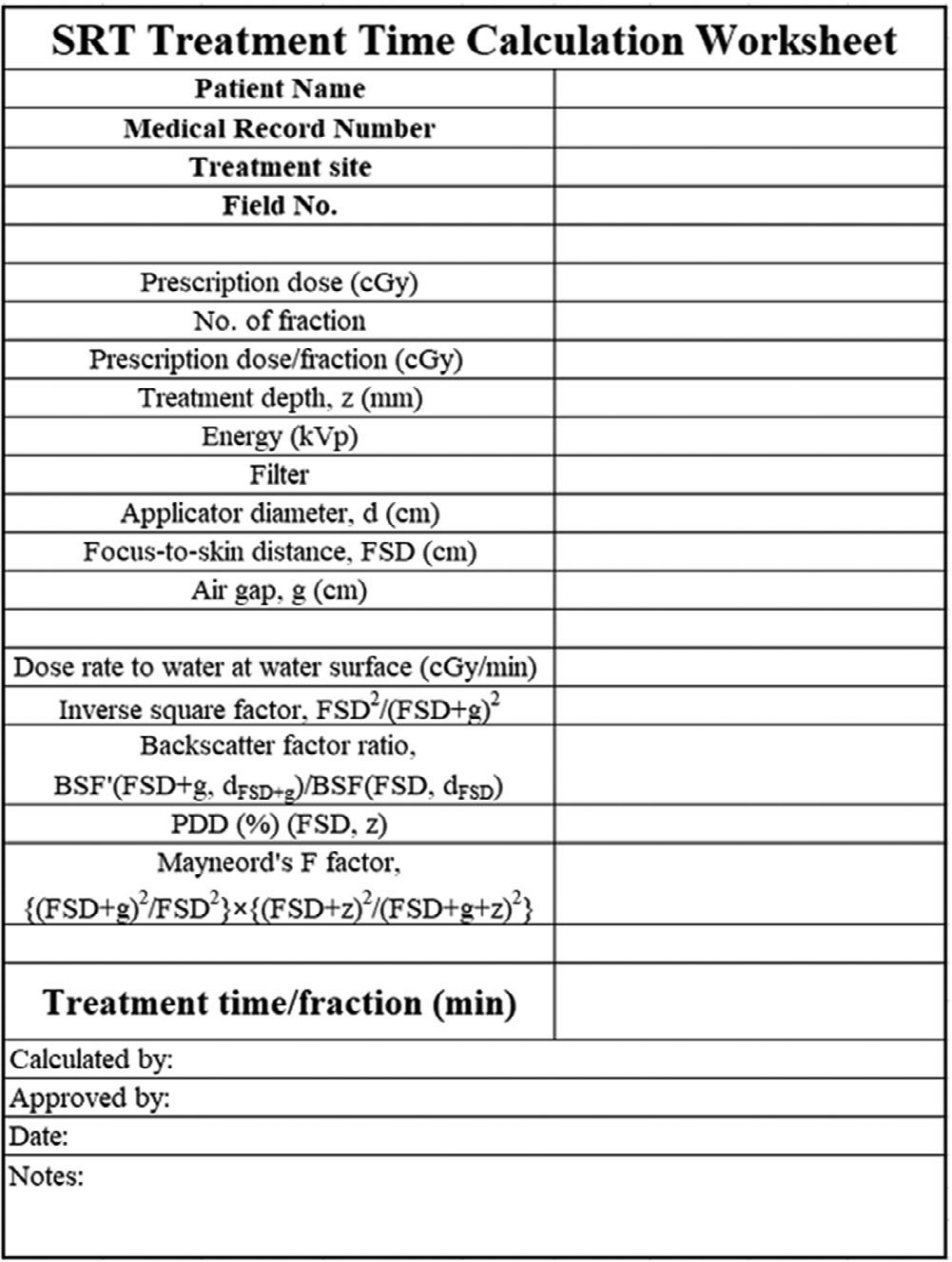
An example of a superficial radiation therapy (SRT) treatment time calculation worksheet

**SRT lookup table**

Alternatively, a lookup table can be created (Figure 5) for clinical use. From the lookup table, treatment time is determined based on prescription dose per fraction, energy, and applicator size as well as prescription depth if applicable. Note that the lookup tables shown in Figure 5 are for illustrative purposes only and not intended for clinical use. Each clinic must generate its own lookup table tailored to its specific SRT system, and the table must be updated promptly following any changes in output or system adjustments.

**FIGURE 5:**
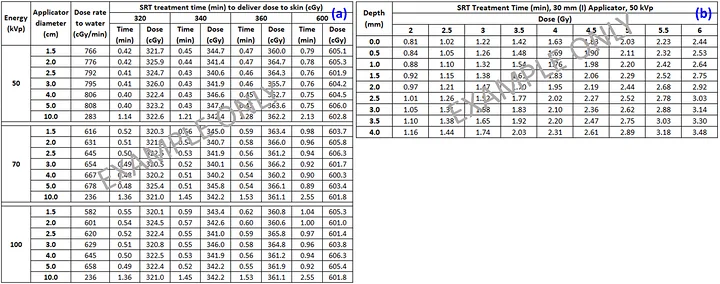
Examples of superficial radiation therapy (SRT) lookup tables created for (a) an SRT‐100 unit and (b) a RADiant Aura unit

**SRT TPS**

With the recent US Food and Drug Administration (FDA) clearance of the Xstrahl XBeam, a TPS dedicated to SRT is available for Xstrahl units. Appropriate commissioning and validation of the XBeam must be completed prior to clinical use.

**Commercial or in‐house software**

The use of commercial or in‐house software is another option. Commercially available software packages (e.g., Radformation ClearCalc) support treatment time calculations for SRT units. Additionally, in‐house software can be developed for clinical use. Appropriate commissioning and validation of such software must be completed prior to clinical implementation.

**MU‐based systems: Xstrahl 150, Xstrahl 200 and Xstrahl 300 units**

Users can select MU‐based calibration for Xstrahl 150, Xstrahl 200 and Xstrahl 300 units (Table 2).^40^, ^41^, ^42^ When MU‐based calibration is selected, the dose rate to water at the water surface, ${\dot{D}}_{w,z=0}$, is specified in cGy/MU and the MU is calculated using Equation (1). The alternative methods described in Section 6.1.4 may also be used for MU calculations where applicable.

#### 6.1.5 Treatment delivery parameter entry

Upon completion of the treatment time or MU calculation, all treatment delivery parameters (prescription dose, number of fractions, frequency of treatment, energy/filter, applicator size, treatment time/MU per fraction, and so on) must be accurately entered or selected in the treatment unit's CI according to the approved treatment plan. When an RO‐EMR system is integrated with the CI, patient information and treatment delivery parameters should transfer automatically. If the RO‐EMR system is not connected to the CI, all patient and treatment data must be manually entered into the RO‐EMR. Relevant checklists for RO‐EMR preparation can be found in Table 5 of MPPG 11.a.^39^

#### 6.1.6 Initial plan/chart review

It is recommended that an initial plan or chart review be independently completed by a QMP or a properly trained individual prior to the first treatment fraction.^39^ Treatment delivery parameters in the treatment plan document must be checked against simulation notes, photos, and the prescription. Treatment time or MUs, calculated using one of the methods described in Section 6.1.4, must be verified by another individual independently. Additionally, treatment delivery parameters saved within CI must be verified. Similarly, when an RO‐EMR system is utilized, patient information and treatment delivery parameters in the system must also be verified. Plan/chart check items pertinent to SRT treatment can be adapted from Table 8 of MPPG 11.a.^39^

#### 6.1.7 Treatment

Prior to treatment delivery, the parameters saved in the CI (e.g., prescription dose, energy/filter paring(s), applicator size and treatment time/MU) must be cross‐checked against the values documented in the signed treatment plan. Unless explicitly planned, air gaps between the applicator and the patient's skin should be minimized during setup to ensure the intended dose is delivered.^24^ Following treatment delivery, a treatment summary report detailing delivered parameters must be reviewed and documented in the patient records or the RO‐EMR system.

### 6.2 Weekly chart review

A weekly chart review by a QMP or a properly trained individual is recommended. The weekly check should include, but is not limited to, treatment site, treatment time/MU calculation, delivered dose to date, number of fractions delivered, dose tracking and any changes to the prescription or plan document since the last check. Example weekly chart review items are listed in Table 10 of MPPG 11.a.^39^

### 6.3 EOT chart review

An EOT chart review must be completed by a QMP or a properly trained individual within five business days of the patient's last delivered fraction.^39^ The EOT review should include, but is not limited to, verification of treatment site, comparison of total delivered dose to the prescribed dose, confirmation of the number of fractions delivered, and validation of all required signatures.^39^

### 6.4 Procedure codes

Relevant procedure codes for SRT treatment, at the time of this writing, are tabulated in Table 4. For additional reference, users may consult the Radiation Oncology Coding Resource published by the American Society for Radiation Oncology (ASTRO), the Current Procedural Terminology Professional published by the American Medical Association and the Revenue Cycle Coding Strategies.

**TABLE 4:** SRT procedure code and description

Procedure	Code	Description
Continuing medical physics consultation	77336	Weekly chart reviews
Simulation and planning	77436	Simulation and basic dosimetry calculation per course
Treatment delivery	77437	Treatment delivery per lesion/target per day
Ultrasonic image guidance	77439	Ultrasonic image guidance per course
Office or other outpatient visit	99202–99205	Evaluation and management of a new patient
	99212–99215	Evaluation and management of an established patient

Abbreviations: SRT, superficial radiation therapy

## 7 REGULATORY REQUIREMENTS AND RADIATION SAFETY

Ownership of a superficial x‐ray unit in a medical facility typically does not require a specific national license.^43^ However, state governments regulate the registration, installation, and safe operations of x‐ray machines.^43^ As these requirements vary by state,^43^ and additional local standards may apply specifically to superficial x‐ray units, it is essential for facilities to strictly adhere to their state and local regulations.

### 7.1 Machine registration

Prior to initiating patient treatments, all superficial x‐ray units must be registered with the relevant state agency. Figure 6 provides a summary of common state regulatory requirements for x‐ray machines. The registration process typically involves several steps. First, the facility must submit a shielding report and associated documentation, prepared by a QMP or a health physicist, to the state agency. Second, the facility registers the machine by submitting a registration form along with the required fee to the state agency. Third, the manufacturer responsible for installing the machine submits FDA form 2579 and any additional state‐specific notification. Fourth, a QMP conducts a comprehensive radiation survey, and submits resulting documentation to the state agency for review. Fifth, a licensed state inspector performs a full inspection of the unit and submits the findings to the state agency. Finally, following machine installation, acceptance testing (where applicable) and commissioning must be completed.

**FIGURE 6:**
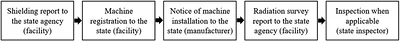
Typical state regulatory requirements for x‐ray machines

### 7.2 Shielding and radiation survey

No specific federal regulations exist regarding shielding design or requirements for x‐ray machines, but state regulations often apply. The facility's shielding design must ensure that radiation exposure for both personnel and the public remains well within the regulatory limits established by state mandates. In the US, shielding calculations for x‐ray machines are typically performed following the recommendations and methodology set forth by National Council on Radiation Protection and Measurements (NCRP) Reports No. 49 and No. 147.^44^, ^45^ Additionally, IAEA Safety Reports Series No. 47 provides relevant information for superficial x‐ray units.^46^ Practical shielding techniques are also covered in a book by Martin, M and Patton, H M.^47^

A vault housing an SRT unit requires structural shielding.^46^ The protection requirement must be based on the highest beam energy intended for clinical use.^46^ SRT units, especially mobile ones, lack isocentric mountings and can potentially irradiate in any direction.^46^ Therefore, with few exceptions, all walls, the floor, and the ceiling must be considered as primary barriers.^46^ The unit must be positioned such that the primary x‐ray beam is never directed at the room door or viewing window during operation.^46^

Following construction and installation of the superficial x‐ray unit, a QMP must conduct a post‐installation radiation survey using a calibrated survey meter. The survey must be performed in full‐scatter conditions with a large phantom, using the highest beam energy and the largest applicator available for clinical use. Leakage radiation measurements are also crucial during this survey,^48^ as these levels will not be routinely checked throughout the unit's lifetime. Prior to clinical use, radiation survey results must demonstrate compliance with the established shielding design goals. The QMP's written report must detail exposure rates in all adjacent occupied areas. For detailed recommendations on conducting radiation surveys, shielding report contents, and suitable survey instruments, one can refer to NCRP Report No. 49.^44^ Additionally, state regulations may mandate that facilities possess calibrated survey meters.

### 7.3 Personnel monitoring

Applicable state regulations dictate whether personal monitoring is required for SRT radiation workers. Implementing active monitoring and periodically reviewing received doses are recommended best practices, regardless of specific regulatory mandates.

### 7.4 Radiation signs and posting

Posting of radiation area signage may be required by state regulations. Additionally, regulations may mandate radiation indicator lights and door interlocks.

### 7.5 Periodic spot checks

State regulations may impose specific requirements for periodic spot checks. The routine QA tests outlined in Table 3 often fulfill these requirements, but the QMP is responsible for ensuring full compliance with all applicable state mandates.

### 7.6 Machine operation

The operation of an SRT unit varies by clinic setting. In a radiation oncology clinic, an RTT typically operates the unit. In a dermatology clinic, either a licensed dermatologist or an RTT performs the procedure. Regardless of the setting, facilities must consult state regulations for specific operational requirements.

### 7.7 Education, licensure, and training of staff

All personnel involved in an SRT program must hold the appropriate degree for their specialty. State regulations may require personnel to be board‐certified by the relevant specialty board. These boards may include the American Board of Radiology, the American Board of Medical Physics, the Medical Dosimetry Certification Board, or the American Registry of Radiology Technologists.

State regulations may also outline licensure requirements for SRT team members (physicians, QMPs and RTTs). If employed in a state requiring licensure (e.g., FL, HI, NY, or TX), these team members must be appropriately licensed. For example, a QMP who provides physics services in multiple states needs to obtain the required licenses or register with each state, as qualification requirements for licensure or registration can vary significantly by jurisdiction.

Prior to initiating patient treatment, all personnel involved in the SRT program must successfully complete comprehensive vendor‐supported on‐site training. This training curriculum must encompass the treatment unit, operational procedures including applicator setup, lead shield utilization, and emergency response protocols. Training materials must be readily accessible at the treatment console, including a prominently displayed copy of emergency procedures. While some states or hospitals may offer general radiation safety courses for credentialing or continuing education, annual in‐service education and training specifically focusing on SRT safety and emergency protocols is highly recommended for all SRT program staff. This annual training can be effectively delivered by a QMP, an RSO or a qualified staff member, as referenced in Section 5.3.3.

## 8 CLINICAL PRACTICE RECOMMENDATIONS

### 8.1 Professional societies

Several professional society reports have addressed practice guidelines for SRT utilizing low‐energy kV x‐rays. These reports encompass, but are not limited to, superficial x‐rays. Early codes of practice from the IPEMB and the IAEA Technical Reports Series No. 398 recommended procedures for reference dose calibration for low‐energy kV x‐ray beams.^9^, ^10^, ^11^ Subsequently, the AAPM TG‐61 established a widely adopted air kerma based protocol (in‐air method) for reference dosimetry.^8^ The recent Swiss Society of Radiobiology and Medical Physics recommendations No. 9 also covers reference dosimetry and recommended detectors.^49^ QC and QA for superficial x‐ray units are addressed in the 2008 IAEA report, the more recent IPEM Repport 81 (2nd ed.) and COMP report.^12^, ^13^, ^34^

Clinical practice guidelines for clinicians using SRT are limited. While ASTRO, the American Academy of Dermatology Association and the National Comprehensive Cancer Network have issued guidelines for treatment of NMSC, there are no specific guidelines dedicated solely to SRT. In 2019, Nestor et al published consensus guidelines on SRT for treatment of NMSC and keloids, including dose fractionation schemes tailored to treatment areas and clinical considerations.^50^ Recently, guidelines for the appropriate utilization of SRT were proposed by Zemtsov et al.^51^ Additionally, Sensus Healthcare provides customers with guidelines regarding treatment margins, kV energy selection and fractionation schemes for various pathologies.

### 8.2 Manufacturers

Medical devices marketed and sold in the US are regulated by the FDA's Center for Devices and Radiological Health, an agency within the Department of Health and Human Services. Consequently, manufacturers must adhere to Title 21 of the Code of Federal Regulations, Parts 800–1299, which govern medical device design, clinical evaluation, manufacturing, packaging, labeling and post market surveillance. Under service contracts, medical device vendors are obligated to promptly notify users of critical issues, system/software upgrades, or scheduled maintenance. In turn, clinical users must report machine malfunctions and any other issues to the vendor, providing necessary data and system access for resolution. Furthermore, users must maintain up‐to‐date instructions for use to ensure compliance with standardized operation, maintenance, and storage procedures.

## 9 CONCLUSIONS

MPPG 18.a provides practice guidance and recommendations for establishing and maintaining SRT programs in dermatology and radiation oncology clinics. The expert recommendations in this report are intended to assist SRT teams in meeting and adhering to minimum clinical practice standards.

## AUTHOR CONTRIBUTIONS

This guideline was reviewed and updated by the MPPG 18.a Working Group of the Professional Council of the AAPM. Each author reviewed recent literature on the topic, offered opinions on and language for the guideline. They also reviewed and applied comments from the full AAPM membership to the document.

## CONFLICT OF INTEREST STATEMENT

The members of MPPG 18.a listed below attest that they have no potential Conflicts of Interest related to the subject matter or materials presented in this document.

Yongsook C. Lee
Seng Boh Lim
Peter M. Mondalek
David G. Saez
Ren‐Dih Sheu
Gerald A. White
Strahinja Stojadinovic

The member of MPPG 18.a listed below discloses the following potential Conflict(s) of Interest related to the subject matter or materials presented in this document.

Sergio O´Reilly,–subcontracted by Sensus Healthcare for commissioning/calibration of SRT units.
